# Unqualified Medical Practice

**Published:** 1910-12-03

**Authors:** 


### The Hospital

A JOURNAL OF

**The Medical Sciences and Hospital Administration.**

NEW SERIES. No. 199, VOL. VIII. [No. 1271, VOL. XLIX.] SATURDAY, DECEMBER 3, 1910.

### UNQUALIFIED MEDICAL PRACTICE.

It is recorded that in the peaceable reign of James I. a warrant was issued for the apprehension of all quacks in order that they might be examined by the Censors of the College of Physicians; and on that occasion several mountebanks, water tasters, ague charmers, and vendors of nostrums were fined, imprisoned, and banished. Similar measures were adopted in the reign of William III., but between then and now offenders of this nature have rarely, if ever, been treated with such wholesome severity. It is true that quackery has been so thoroughly exposed that one would think there was hardly "a man in the world," to use the words of Sir Richard Steele, "so ignorant as not to know that the ordinary quack doctors . . . are to a man impostors and murders," yet, still quoting the great essayist, "such is the credulity of the vulgar, and the impudence of these professors, that the affair still goes on, and new promises of what was never done before are made every day." The most recent exposure of quackery consists of a collection of facts furnished to the Privy Council by sixteen hundred medical officers and recorded in a Government publication. Since the extent to which irregular medical practice is carried on has been brought to the notice of the Government there is at least some ground for hope that, in the interest of the public health, steps will be taken to introduce legislation to put an end to the evil, or, at any rate, to restrict it. The report in question deals with a variety of forms of quackery, and it will surprise many to learn that in some rural districts "wise women" still do a certain amount of medical and surgical practice, employing methods "often of a disgusting character." Belief in witchcraft is still held by a few people, and in Wales and the West Riding of Yorkshire "water doctors" continue to carry on a lucrative business, their methods being no doubt similar to those of Cameron, which were described in THE HOSPITAL of July 9, 1910 (page 437). It is surely erroneous, by the way, to describe the persons who carry on this objectionable practice as "chemists," a title which is restricted to persons registered under the Pharmacy Act. Indeed, the term "chemist," as used in the Report, seems to apply alike to pharmacists and to those who pretend to be such, and the legitimate users of the title are likely to suffer in consequence. The Report states that prescribing by chemists is so common "as to be practically universal throughout the country," with the reservation that "qualified and unqualified chemists are not always distinguished, but it would seem that the latter prescribe more recklessly than the former." While in the main such prescribers confine their attention to the so-called minor ailments, and advise patients to consult a doctor in the more difficult cases, the evidence shows that a large amount of prescribing in what eventually proves to be disease of a graver character also takes place. As to "Herbalists," their trade appears to be a lucrative one, and in several parts they are stated to have a higher popularity than qualified medical men; some of these impudent fellows actually warn the public against quacks. Herbalists are concerned in the spread of epidemics of infectious diseases and some of them safeguard themselves by giving an indefinite certificate—*e.g.* "The case is of a diphtheritic, or typhoid, etc., nature." As to "Bonesetters," their manipulations appear sometimes to occasion real injury, when the injury professed to be treated has actually been suppositious. Their lack of knowledge prevents them from distinguishing tuberculous bone disease from an ordinary dislocation, and a large number of medical officers of health refer to the disastrous results to individuals in such cases. With regard to "Dentists," it is evident that the practice of dental surgery by unqualified persons is assuming larger proportions, and the public is frequently misled as to the qualifications of unqualified dentists. Practice by Christian Scientists and Faith Healers is increasing in several districts, and a somewhat kindred practice—prescribing while the patient is under hypnotic influence—exists in the West Riding of Yorkshire and Durham. Perhaps one of the most important parts of the Report is that which deals with proprietary medicines, the use of which is extremely widespread throughout the country. The evidence furnished in the Report against secret remedies, however, is by no means so convincing as that contained in the Report of the Australian Royal Commission, referred to in THE HOSPITAL of January 4, 1908. One of the worst effects of proprietary medicines is shown in the enormous amount of self-drugging practised, which is rendered so easy by the ready access which everyone may have to all kinds of drugs in tablet, liquid, or pilule form. This, as the Report states, is a habit which has increased enormously during the past few years. The suggestions made by medical officers of health as to proprietary medicines and infant foods are as follows: (1) The composition of all advertised remedies should be stated; (2) their formulas should be determined by Government analysis at the vendor's expense; (3) the sale of headache-powders and such-like drugs should be prohibited; (4) the sale of infants' foods should be regulated. As to the fourth recommendation, it has already been pointed out in THE HOSPITAL that while the law requires that certain conditions shall be fulfilled by manufacturers of food for sheep, horses, cows, and chickens, it places no restrictions on the manufacture of food for babies.

